# Collaborative Retrieval Practice Reduces Mind-Wandering During Learning

**DOI:** 10.1027/1618-3169/a000589

**Published:** 2023-10-13

**Authors:** Alexander G. Knopps, Kathryn T. Wissman

**Affiliations:** ^1^ Department Psychology, North Dakota State University, Fargo, ND, USA

**Keywords:** collaborative learning, mind-wandering, retrieval practice

## Abstract

**Abstract:** Research has shown engaging in retrieval practice can reduce the frequency of mind-wandering. However, no prior research has examined how engaging in collaborative (as compared to individual) retrieval practice impacts mind-wandering during learning. In the current experiment, participants were asked to study a list of words, followed by retrieval practice that either occurred collaboratively (as a dyad) or individually. During retrieval practice, participants provided self-reports as to whether they were on task or off task. Following retrieval practice, all participants completed an individual final test. Of greatest interest, the results showed that engaging in collaborative retrieval practice decreased the frequency of mind-wandering during learning. In addition, and consistent with prior collaborative learning research, collaborative inhibition during practice and postcollaborative benefits on the final test were observed. The current results provide the first demonstration of an additional benefit to using collaborative retrieval practice: This technique reduces the frequency of mind-wandering.


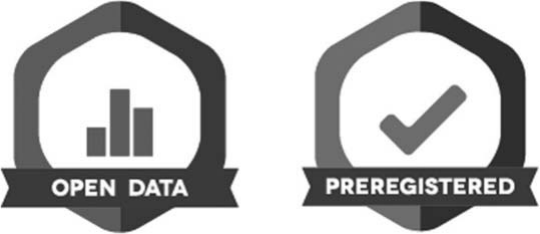


A typical college student is expected to learn (and remember) a lot of information, which inevitably requires paying attention on behalf of the student. Of course, a student cannot be expected to pay attention all the time. Mind-wandering is a phenomenon described as a shift of attention away from an intended or primary task (for a seminal review, Smallwood & Schooler, 2006; also see, Pachai et al., 2016; Randall et al., 2014) and is a commonly occurring experience (Kane et al., 2017; Killingsworth & Gilbert, 2010; Seli et al., 2018; Smith et al. 2018). Although mind-wandering can be beneficial in some situations (e.g., for creativity, Baird et al., 2012), a majority of research indicates negative consequences of mind-wandering (for cognitive functioning, Cheyne et al., 2006; for reading comprehension, Unsworth & McMillan, 2013; for memory retrieval, Martin et al., 2018). As such, one way to support student learning is finding ways to reduce the frequency of mind-wandering. The current research investigates a novel technique that may reduce the likelihood of mind-wandering during learning: collaborative retrieval practice.

Mind-wandering is a common experience, with research estimating that individuals mind-wander between 30% and 50% on any given day (Killingsworth & Gilbert, 2010). Astoundingly, research conducted in authentic classroom settings suggests that students may be distracted every 5–6 min (Judd & Kennedy, 2011; Rosen et al., 2013). Given the frequency at which students tend to mind-wander, research has investigated how mind-wandering impacts the recall and memory of information (Risko et al., 2012; Wammes et al., 2016). Unfortunately, research has found that mind-wandering can negatively impact understanding of to-be-learned information (Unsworth & McMillan, 2013; for a review, Szpunar, Moulton, & Schacter, 2013). As such, research has started examining different techniques that can be used to *reduce* the likelihood of mind-wandering (for testing during encoding, Szpunar, Khan, & Schacter, 2013; for retrieval practice, Peterson & Wissman, 2020; for interleaving, Eglington & Kang, 2017; for spaced practice, Metcalfe & Xu, 2016). For example, Peterson and Wissman (2020) explored how mind-wandering rates were impacted when participants engaged in retrieval practice versus restudy. Across two experiments, participants were asked to learn categorical word lists. Critically, after encoding, participants engaged in either retrieval practice or restudy to further learn the word lists. Of interest for current purposes, during this review phase, all participants responded to four mind-wandering probes. Following review, all participants completed a final recall test. In both experiments, the results showed that participants reported significantly less mind-wandering in the retrieval practice group compared to the restudy group, providing evidence that (individual) retrieval practice testing reduces the frequency of mind-wandering during learning.

Although research has shown individual retrieval practice can reduce mind-wandering during learning, it is an open question as to whether *collaborative* retrieval practice will have the same effect. Exploring how collaborative retrieval practice impacts mind-wandering rates has important implications for applied purposes. Indeed, across many academic content domains, students are often required to learn in collaborative environments. Research indicates that faculty increasingly incorporate collaborative learning into their classrooms (National Research Council (U.S.), 2012), with outcomes suggesting that working in small groups is beneficial (for an overview, Slavin, 2012; for a meta-analysis, Kyndt et al., 2013) while also providing the opportunity for building connection amongst peers (Kuh, 2001; Summers et al., 2005). Of course, the term “collaboration” can be operationalized in various ways. For example, research has examined the effects of collaborative encoding (Barber et al., 2010), collaborative learning activities (for recall, Harris et al., 2008; for think–pair–share, Deshpande et al., 2020; for jigsaw groups, Button et al., 2021), and collaborative exams (LoGiudice et al., 2023). Survey research has also revealed that learners report studying in groups (Geller et al., 2018; Hartwig & Dunlosky, 2012) and that they specifically use retrieval practice as a learning technique in these collaborative settings (Wissman & Rawson, 2016). The current research involves collaboration during learning, with a focus on the use of collaborative retrieval practice.

In a typical collaborative learning design, participants are first asked to individually study some type of to-be-learned material. Participants then engage in retrieval practice, which often involves more than one recall trial and occurs either individually or collaboratively. After some delay, participants complete an individual, final test. The two most common findings are that recall during retrieval practice is lower for participants working in a group versus alone (*collaborative inhibition*; Basden et al., 1997; Weldon & Bellinger, 1997), whereas recall on the final test is greater for participants who previously worked in a group versus alone (*postcollaborative benefits*; Blumen & Rajaram, 2008; Wissman & Rawson, 2015). The finding that collaborative retrieval practice can enhance subsequent, individual memory suggests this is an effective study technique for students to use. More generally, research indicates that faculty and students use collaboration in educational settings and that collaborative retrieval practice can have a positive impact on memory; another unexplored benefit of collaborative retrieval practice is it may help students stay on task.

Existing theoretical accounts from mind-wandering research suggest this technique may reduce mind-wandering rates even further (for an overview of theories, see Pachai et al., 2016). For example, the executive failure hypothesis (McVay & Kane, 2009, 2012; Smallwood, 2013) posits that individuals have a limited number of cognitive resources, so to focus on the primary task, the executive control system attempts to reduce both internal and external distractions; according to this account, mind-wandering occurs when the system fails to inhibit internal distractions. When working in a collaborative setting, one must not only focus on the primary task but also work to coordinate cognitive efforts with social efforts (Grau & Whitebread, 2012), which presumably requires more executive control (as compared to working alone, as would occur with individual retrieval practice). Furthermore, research has shown that when more executive resources are required to maintain focus, the less likely one is to mind-wander (Hawkins et al., 2022; Kane et al., 2017; McVay & Kane, 2009). Another reason collaborative retrieval practice may reduce the frequency of mind-wandering concerns motivation. Research has shown that individuals report greater engagement and motivation when working in a group (Seli et al., 2019) and that higher levels of motivation reduce mind-wandering rates (Kang & Pashler, 2014; Seli et al., 2019). Thus, increased motivation would also suggest the frequency of mind-wandering may be lower when engaging in collaborative versus individual retrieval practice.

The goal of the current research was to evaluate how collaborative retrieval practice impacts the frequency of mind-wandering during learning. Learners were asked to study a list of words and then engage in collaborative or individual retrieval practice. All learners responded to eight mind-wandering probes during retrieval practice by providing self-reports of whether they were on or off task. Following retrieval practice, all learners completed an individual final recall test. Based on prior research and theoretical accounts, we predicted that mind-wandering would be lower for learners engaging in collaborative versus individual retrieval practice.

## Method

### Participants and Design

Undergraduates who participated for course credit (*n* = 106) were randomly assigned to one of two groups: collaborative or individual. Participants were excluded from final analysis due to noncompliance (i.e., responding to fewer than 75% of the mind-wandering prompts; *n* = 5). When there was noncompliance with one (collaborative or nominal) group member, data for the other group member were also excluded (*n* = 5). The final sample included *n* = 96 participants (55% female; average age = 19 years): *n* = 50 in the individual group and *n* = 46 in the collaborative group. The a priori sample size was *n* = 102, with power set to .80 and one-tailed α = .05 to detect a medium effect of *d* = .50 (Faul et al., 2009). This experiment’s pre-registration can be found in Open Science Framework (https://osf.io/u7xpe).

### Materials

Materials included a 48-item word list, comprising six exemplars from eight categories (Van Overschelde et al., 2004). Materials also included mind-wandering probes (Peterson & Wissman, 2020; Unsworth & Robison, 2016), which asked participants “What were you just thinking about?” and provided the following six response options: (1) The current task. (2) My performance on the current task. (3) A memory from the past. (4) Something in the future. (5) Current state of being. (6) Other. Finally, three postexperiment questions (PEQs) asked about participants’ perception of task difficulty (from 1 = *very easy* to 5 = *very difficult*), confidence in staying on task (from 1 = *extremely unconfident* to 5 = *extremely confident*), and motivation to stay on task (from 1 = *very unmotivated* to 5 = *very motivated*).

### Procedure

Prior to beginning the experiment, participants were given a brief overview, which included our interest in examining mind-wandering. Participants were told mind-wandering was a normal phenomenon and asked to respond honestly to the probes presented throughout the experiment (for a similar approach, see Peterson & Wissman, 2020). Participants were also informed they may be asked to work in a group at some point during the experiment (although it was not specified when collaboration would occur).

Phase 1 involved an initial study of the exemplars and was completed individually by all participants. Prior to being shown the exemplars to study, participants were informed that they would be tested on their memory for the words later in the experiment. Exemplars were presented in a random order two times, and participants were given 5 s to study each exemplar. Following Phase 1, participants were told to see the research assistant for further instructions and then informed whether they would work alone or in a group for the next phase. Phase 2 involved recall of the previously studied words and was completed either individually or collaboratively (as a dyad). During this phase, participants were asked to type words into a provided textbox and hit enter after each word. After hitting enter, the word was added to a textbox shown on the screen to remind participants which words they recalled. Participants working individually sat at their own computer to recall the words. Participants working collaboratively sat at one computer to recall the words; one group member was instructed to type in their responses, and they were told to work together however they saw fit. During Recall 1, participants were given 5 min and asked to recall as many words as they could remember. Participants then engaged in Recall 2, which occurred in the same way as Recall 1. All participants were asked to do their best to use the entire time to recall as many words as possible and told that the computer would automatically advance them when time was up. Critically, participants were presented with a total of eight mind-wandering probes across Recalls 1–2. All participants provided individual responses to mind-wandering probes via pencil-and-paper.^1^

Participants were given 10 s to respond to the probe. The mind-wandering probes occurred at four predetermined times during Recall 1 (37 s, 101 s, 156 s, and 268 s) and Recall 2 (79 s, 134 s, 203 s, and 240 s) to ensure that participants in both groups provided mind-wandering ratings at the same time. Following Phase 2, all participants completed an unrelated filler task for 3 min. Phase 3 was completed individually by all participants. During this phase, participants took the final test; they were given 7 min and asked to recall as many words as they could remember. Participants then answered the three PEQs about task experience.

### Scoring

Consistent with mind-wandering literature, responses to mind-wandering probes were categorized as “on task” if a participant responded with “The current task” or “My performance on the current task”, with all other responses coded as “off task”; for interested readers, the breakdown of all six response categories can be accessed via OSF (https://osf.io/52v8w).

Consistent with collaborative memory literature, performance during Phase 2 compares collaborative recall (*n* = 25 groups) versus nominal recall (*n* = 23 groups), with nominal groups being formed by combing the protocols (i.e., pooling nonredundant items) of two participants in the individual group who completed the experiment on approximately the same day. For performance during Phase 3, outcomes are reported at the individual level.

## Results

**Figure 1:**
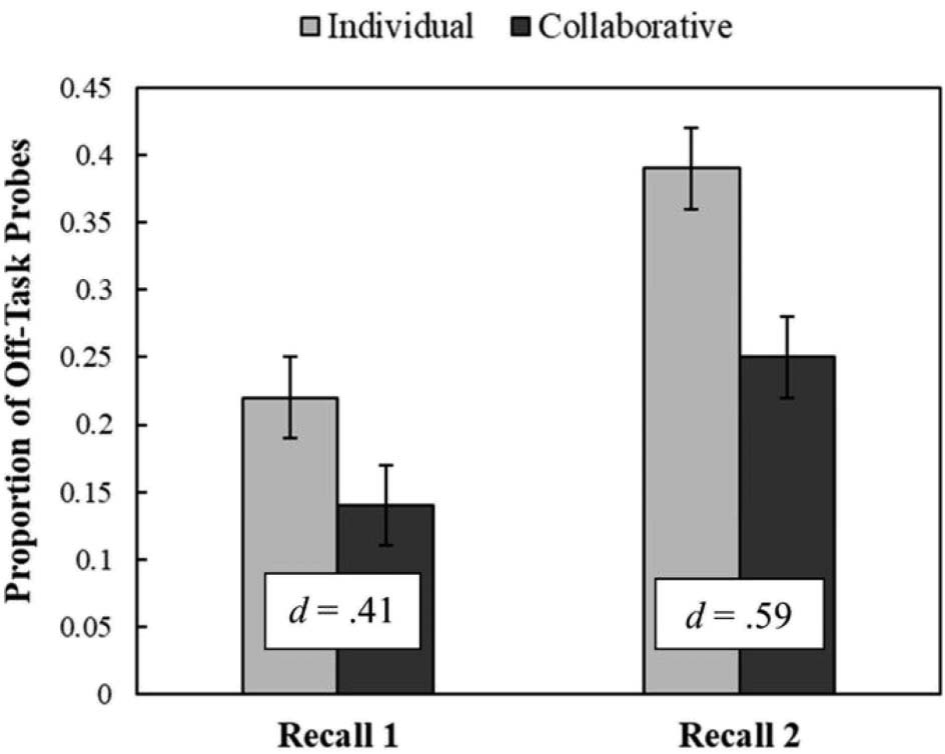
Mind-wandering rates during Recall 1 and Recall 2. Proportion of mind-wandering probes eliciting off-task thinking responses. Error bars report standard error of the mean. Cohen’s *d* effect size is reported in the box.

**Figure 2:**
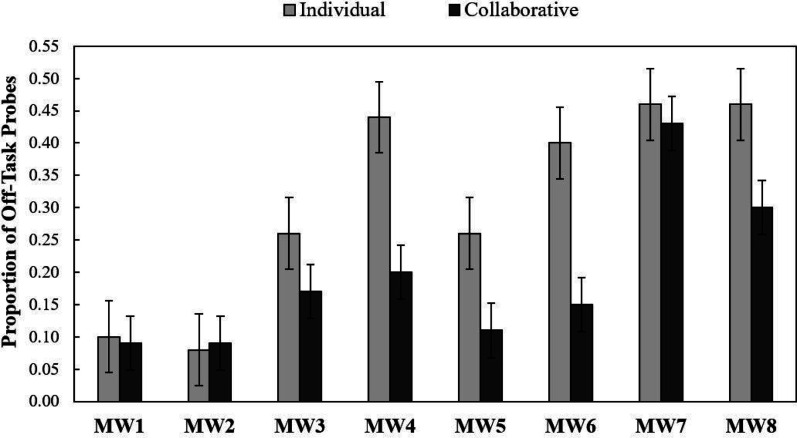
Mind-wandering rates across all eight probes. Proportion of mind-wandering probes eliciting off-task responses. MW refers to the mind-wandering probe number. Error bars report standard error of the mean.

Figure 1 provides the proportion of mind-wandering probes eliciting off-task thinking for the collaborative versus individual group during Recall 1 (left set of bars) and Recall 2 (right set of bars). The results indicate that participants in the collaborative versus individual group were less likely to report being off task during both Recall 1 [*t*(94) = 2.02, *p* = .023, *d* = .41] and Recall 2 [*t*(94) = 2.90, *p* = .002, *d* = .59]. These outcomes provide novel evidence that collaborative retrieval practice reduces the frequency of mind-wandering during learning. For exploratory purposes, we conducted a 2 (group: collaborative vs. individual) × 8 (mind-wandering probe: 1 vs. 2 vs. 3 vs. 4 vs. 5 vs. 6 vs. 7 vs. 8) repeated-measures ANOVA^2^ to provide a finer-grained analysis of mind-wandering rates across all eight probes for both groups (see Figure 2).

Outcomes revealed a significant main effect of group [*F*(5.8, 543.7) = 10.47, *p* < .001, η_*p*_^2^ = .100] and mind-wandering probes [*F*(1, 94) = 8.93, *p* = .004, η_*p*_^2^ = .087]; the interaction was not significant (*p* = .157). These outcomes indicate mind-wandering increased across the experiment (for similar findings, see Seli et al., 2018) and the likelihood of mind-wandering was reduced for participants in the collaborative versus individual group.

**Figure 3:**
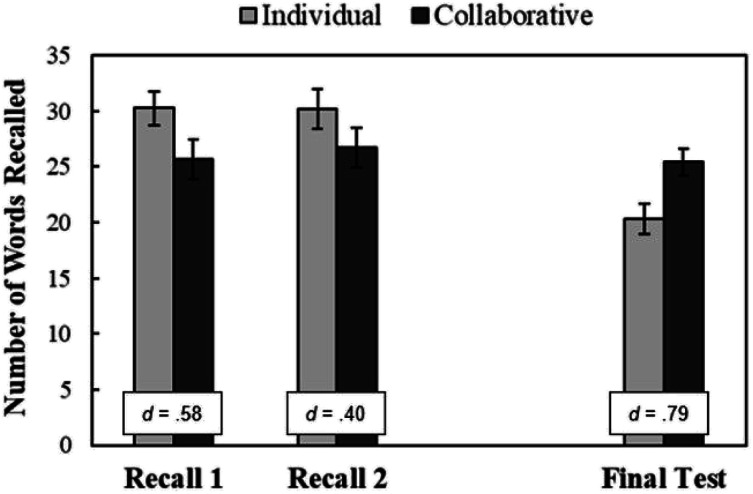
Words recalled during Recall 1, Recall 2, and final test. The number of exemplars recalled during Phase 2 and Phase 3. Error bars report standard error of the mean. Cohen’s *d* effect size is reported in the box.

Concerning recall, Figure 3 provides the number of words recalled during retrieval practice in Phase 2 (Recall 1 and Recall 2; see left two sets of bars) and on the final test in Phase 3 (see right set of bars) for the collaborative versus individual group. The results revealed that recall during practice was lower for participants in the collaborative versus individual group during Recall 1 [*t*(46) = 2.01, *p* = .025, *d* = .58] and Recall 2 [*t*(46) = 1.38, *p* = .088, *d* = .40]. These outcomes provide evidence of collaborative inhibition during practice, which replicates prior research (Blumen & Rajaram, 2008; Weldon & Bellinger, 1997). The results also showed that recall on the final test was significantly greater for the participants who previously engaged in collaborative (*M* = 25, *SE* = 1) versus individual (*M* = 20, *SE* = 1) retrieval practice, *t*(93) = 2.75, *p* = .004, *d* = .79. This outcome provides evidence of postcollaborative benefits, also replicating prior research (Blumen & Rajaram, 2008; Wissman & Rawson, 2015).

Finally, we examined responses to the three PEQs (see Table 1). Participants in the collaborative versus individual group reported significantly less difficulty in staying on task, *t*(94) = 2.31, *p* = .012, *d* = .47. Responses to PEQs regarding confidence and motivation did not differ across the two groups (*p* = .065 and *p* = .358, respectively).

**Table 1:** Participant responses to postexperiment questions (PEQ)

PEQ	Collaborative	Individual
Difficulty staying on task	2.4 (0.1)	2.8 (0.1)
Confidence staying on task	3.8 (0.1)	3.5 (0.1)
Motivation to stay on task	3.9 (0.1)	4.0 (0.1)
*Note*. *SE* of the mean is reported in parentheses.		

## Discussion

The current study investigated how engaging in collaborative retrieval practice impacts the frequency of mind-wandering. Outcomes provide the first demonstration that collaborative (compared to individual) retrieval practice significantly reduced the frequency of mind-wandering during learning. Replicating prior collaborative memory research, outcomes also showed a cost of collaboration during practice (i.e., collaborative inhibition), but a benefit to later individual memory (i.e., postcollaborative benefits). The current outcomes suggest an additional, and novel, benefit to using collaborative retrieval practice as a learning technique – it helps reduce mind-wandering.

Why might collaborative retrieval practice reduce the frequency of mind-wandering? As briefly discussed earlier, the executive failure hypothesis suggests mind-wandering occurs when the control system fails to inhibit internal distractions (McVay & Kane, 2009), and we posited that coordinating recall efforts with another individual during retrieval practice may help reduce the likelihood of such internal distractions. Complementary to this account is research showing that mind-wandering requires executive resources, and when these resources are utilized elsewhere (for example, in a collaborative context), mind-wandering rates decrease (Smallwood, 2010). If working collaboratively is a more demanding task that requires more effort and executive control on the part of each individual, this may in turn help decrease the likelihood of internal distractions. Further research that systematically examines underlying mechanisms that contribute to reductions in mind-wandering rates in collaborative settings is an important direction for future research.

In the current research, time on task during learning was equated to ensure that all participants responded to the same number of mind-wandering probes at the same time. An interesting direction for future research would be to explore the efficiency of working collaboratively versus individually, and how this may impact mind-wandering rates. For example, initial research suggests it takes longer to achieve similar levels of performance when engaging collaborative versus individual retrieval practice (Wissman & Rawson, 2018, Experiments 2 and 3). In terms of mind-wandering, off-task thinking may become more likely as time elapses if working alone is simply more efficient.^3^ To explore this, participants could engage in self-paced (collaborative vs. individual) retrieval practice during which they respond to self-caught mind-wandering probes. Although beyond the scope of the current study, exploring the relationship between collaborative versus individual retrieval practice and the frequency of mind-wandering from an efficiency standpoint would be informative.

The finding that collaborative retrieval practice reduces mind-wandering has promising implications for educational purposes. An important next step will be to investigate this effect in more authentic, learning contexts (for example, inside the classroom or out-of-class group study sessions). Indeed, in more real-world settings, there will be an increased likelihood of other external distractions, such as noisy classrooms and electronic devices. In addition, in real-world collaborative settings, learners are more likely to know one another as opposed to being strangers (as was the case with the current study). Interestingly, previous research has shown that collaborative inhibition is attenuated when groups comprise friends (Andersson, 2001; Andersson & Rönnberg, 1995, 1996). One possibility is the likelihood of mind-wandering during collaboration may also be affected by group composition. Investigating how collaborative retrieval practice impacts the likelihood of mind-wandering in more authentic, educational settings is another fruitful avenue for future research.

Although not of primary interest, it is worth highlighting that recall outcomes replicate prior collaborative memory research such that collaborative inhibition was observed during practice and postcollaborative benefits were observed on the final test. The latter is particularly important, as most studies have focused on investigating collaborative inhibition with far fewer studies focusing on postcollaborative benefit, which is arguably the more important effect from an applied standpoint as learning and retention are ultimately assessed at the individual level. One interesting idea is to examine if reductions in mind-wandering rates during learning have downstream effects. For example, one could include mind-wandering probes during the final test to determine the extent to which collaborative retrieval practice impacts the likelihood of mind-wandering on later individual tests.

The current study provides novel outcomes to the literature by showing that engaging in collaborative versus individual retrieval practice significantly reduces the likelihood of mind-wandering during learning. Given the importance of replicating novel findings (e.g., Camerer & Wu, 2018; Pashler & Harris, 2012; Roediger et al., 2012; Simons, 2014), future work should replicate and further evaluate the effects observed here, as understanding when and why collaborative retrieval practice decreases mind-wandering rates will have important implications for supporting student learning. Importantly, the current research provides foundational outcomes as a basis for further investigation of how collaborative retrieval practice impacts the frequency of mind-wandering during learning.

## Footnotes

^1^
Mind-wandering probes were recorded on paper and pencil to ensure each participant could respond privately and avoid social comparison effects (Weinstein et al., 2018).

^2^
Sphericity was violated (**ε** = .453), so Greenhouse–Geisser corrected results are reported.

^3^
It is worth noting that at the start of a new recall trial (i.e., Recall 2), the proportion of off-task thinking was significantly greater in the individual group compared to the collaborative group (for Probe 5, *M* = 0.26 vs. *M* = 0.11, *t*(94) = 1.91, *p* = .029, *d* = .39; for Probe 6, *M* = 0.40 versus *M* = 0.15, *t*(94) = 2.78, *p* = .003, *d* = .57).

## Notes

**Authorship:** Alexander G. Knopps: Conceptualization, data curation, formal analysis, investigation, methodology, supervision, validation, visualization, writing – original draft, and writing – review & editing; Kathryn T. Wissman: Conceptualization, data curation, formal analysis, investigation, methodology, project administration, software, supervision, validation, visualization, writing – original draft, and writing – review & editing.

**Open Data:** This experiment’s project materials (raw data) can be found in Open Science Framework at https://osf.io/ns7qx/ (Wissman & Knopps, 2023).

Pre-registration can be found at https://osf.io/u7xpe (Wissman & Knopps, 2021).
